# Supplementary material for: Predictive Biomarkers for Checkpoint Inhibitor-Based Immunotherapy: The Galectin-3 Signature in NSCLCs
Source: Int J Mol Sci. 2019 Mar 31;20(7):1607. doi: 10.3390/ijms20071607 (PMC6479404; doi:10.3390/ijms20071607)
Supplement: Supplementary file 1 [file ijms-20-01607-s001.pdf]

Patients	Tumor Subtype	EGFR gene mutational status (exons 18-19-20-21)	ALK translocations	ROS1 rearrangement	PDL1 (TPS %)
1	SCC	WT	negative	negative	>50%
2	SCC	WT	negative	negative	>50%
3	Adc	WT	negative	negative	>50%
4	Adc	WT	negative	negative	>50%
5	Adc	WT	negative	negative	>50%
6	Adc	WT	negative	negative	>50%
7	Adc	WT	negative	negative	>50%
8	Adc	WT	negative	negative	>50%
9	Adc	WT	negative	negative	>50%
10	Adc	WT	negative	negative	>50%
11	SCC	WT	negative	negative	>50%
12	Adc	WT	negative	negative	>50%
13	SCC	WT	negative	negative	>50%
14	Adc	WT	negative	negative	>50%
15	Adc	WT	negative	negative	>50%
16	Adc	WT	negative	negative	>50%
17	Adc	WT	negative	negative	>50%
18	Adc	WT	negative	negative	>50%
19	Adc	WT	negative	negative	>50%
20	Adc	WT	negative	negative	>50%
21	SCC	WT	negative	negative	>50%
22	Adc	WT	negative	negative	>50%
23	Adc	WT	negative	negative	>50%
24	Adc	WT	negative	negative	>50%
25	Adc	WT	negative	negative	>50%
26	Adc	WT	negative	negative	>50%
27	SCC	WT	negative	negative	>50%
28	Adc	WT	negative	negative	>50%
29	Adc	WT	negative	negative	>50%
30	Adc	WT	negative	negative	>50%

31	Adc	WT	negative	negative	>50%
32	Adc	WT	negative	negative	>50%
33	Adc	WT	negative	negative	>50%
34	Adc	WT	negative	negative	>50%

Legend:

SCC: Squamous Cell Carcinoma;

Adc: Adenocarcinoma

EGFR: epidermal growth factor receptor

WT: wild type

ALK: Anaplastic lymphoma kinase

ROS1: ROS Proto-Oncogene1, Receptor Tyrosine Kinase

TPS: tumor proportion score

PD-L1 immunostaining with score > 50% (PD-L1 tumor expression) is specifically required for starting immunotherapy (Reck M. *et al.* *N. Engl. J. Med.* ***2016,*** *375,1823-1833* . PMID: 27718847)

Supplementary table 2

Fisher's exact test

	**iRECIST**		**TOT**	**p value**
	**CR – PR - SD**	**PD**		
**Negative/Low/Intermediate GAL-3**	13	0	13	> 0.0001
**High GAL-3**	2	19	21	
**TOT**	15	19	34	

Legend.

CR: complete response; PR: partial response; SD: stable disease;

PD: progressive disease.

The two-tailed P value is less than 0.0001
